# Mesenchymal Stromal Cell Dependent Regression of Pulmonary Metastasis from Ewing’s

**DOI:** 10.3389/fped.2014.00044

**Published:** 2014-05-30

**Authors:** Andrea Hayes-Jordan, Yong Xin Wang, Peter Walker, Charles S. Cox

**Affiliations:** ^1^Department of Surgical Oncology, University of Texas MD Anderson Cancer Center, Houston, TX, USA; ^2^University of Texas Medical School at Houston, Houston, TX, USA

**Keywords:** stem cell, Ewings sarcoma, lung metastases, pediatrics, murine model, mesenchymal stromal cells

## Abstract

**Introduction:** Ewing’s sarcoma (ES) is the second most common bone tumor in children. Survival has not improved over the last decade and once pulmonary metastatic disease is present, survival is dismal. Mesenchymal stromal cell (MSC) therapy has shown potential benefit for Kaposi’s sarcoma; however, the role of progenitor cell therapies for cancer remains controversial. MSC treatment of ES or pulmonary metastatic disease has not been demonstrated. We have developed an orthotopic xenograft model of ES in which animals develop spontaneous pulmonary metastases. Within this model, we demonstrate the use of MSCs to target ES lung metastasis.

**Materials and Methods:** Human ES cells were transfected with luciferase and injected into the rib of nude mice. Development of pulmonary metastases was confirmed by imaging. After flow cytometry based characterization, MSCs were injected into the tail vein of nude mice with established local ES tumor or pulmonary metastasis. Mice were treated with intravenous MSCs weekly followed by bioluminescent imaging.

**Results:** The intravenous injection of MSCs in an ES model decreases the volume of pulmonary metastatic lesions; however, no effect on primary chest wall tumor size is observed. Thus verifying the MSC preferential homing to the lung. MSCs are found to “home to” the pulmonary parenchyma and remain engrafted up to 5 days after delivery.

**Discussion:** MSC treatment of ES slows growth of pulmonary metastasis. MSCs have more affinity for pulmonary metastasis and can effect a greater decrease in tumor growth in the lungs compared to the primary tumor site.

## Introduction

Ewing’s sarcoma (ES) is a primitive neuroectodermal tumor and is the second most common bone tumor in children and young adults (ages 5–30) (1). Current multimodal therapy for localized disease consisting of tumor resection with/without radiation and concordant multidrug chemotherapy, still fails 20–40% of the time resulting in metastatic disease. Metastatic ES continues to have a dismal prognosis with fewer than 20% of patients surviving at 5 years. Despite multiple attempts to develop novel therapies including total body irradiation with autologous bone marrow transplantation (2) and induction with highly toxic chemotherapeutic regimens (3), no improvement in the mortality associated with metastatic disease (approaching 90%) has been observed for over 40 years (4).

A growing amount of preclinical research has been completed investigating the potential role of bone marrow derived mesenchymal stromal cell (MSC) therapy for the treatment multiple forms of cancer. By definition, MSCs are multipotent and have the capacity for self renewal (5) making them ideal candidates for novel therapeutic strategies. The intravenous injection of MSCs has been associated with migration toward the site of tumor inflammation with increased levels of engraftment (6). In non-tumorigenic animal models, MSCs have also been found to preferentially migrate to the lung after intravenous injection (7). Pulmonary passage is a major obstacle for intravenous stem cell delivery: the pulmonary first-pass effect. The observed increased engraftment in the lungs makes MSCs attractive as vehicles for the delivery of anti tumorigenic proteins to pulmonary sites.

Preliminary research using an ES model has shown the successful intravenous delivery of MSCs transfected with a gene for the anti tumorigenic protein interleukin 12 (IL-12). The transplanted MSCs were found to engraft at tumor sites and increase local IL 12 production leading to a decrease in tumor burden (8). While such initial studies have shown great potential, limited research has been completed to investigate the role of progenitor cell therapies for ES. There are no studies currently published to evaluate the potential role of MSCs in ES lung metastasis.

The design of future studies to investigate the potential use of MSC therapy for ES requires the development of tested animal models. Therefore, we evaluated the effect of intravenous MSC injection on both primary and metastatic tumor sites using a novel ES model (9). We hypothesize that the transplanted MSCs will engraft in the lung and decrease pulmonary metastasis.

## Experimental Design and Methods

### *In* *vivo*

All procedures were approved by the institutional animal care and use committee and were consistent with the National Institutes of Health’s Guide for the Care and Use of 13 Laboratory Animals (HSC-AWC-07-031).

TC71 human ES cells transfected with a luciferase reporter were injected in the rib of nude female mice. As previously described, our model is associated with a 60% incidence of chest wall tumors alone, 30% incidence of pulmonary metastasis alone, and 10% incidence of synchronous chest wall and pulmonary metastatic tumors (9). Next, quantum dot labeled rat MSCs were injected via the tail vein at 1, 2, and 3 weeks after ES implantation for a total of three doses. Then, the mice were harvested at 1, 2, and 3 weeks after completion of the MSC injections. Chest wall and pulmonary metastasis tumor burden as well as quantum dot labeled MSC location and burden were then measured.

### Orthotopic chest wall model

The ES model has been described in a previous publication (9). Briefly, approximately 8-week old, 25 g, female athymic nude (nu/nu) mice were obtained from the National Cancer Institute (Frederick, MD, USA). A total of 500,000 TC71 cells were suspended in 20 μL of phosphate buffered saline (PBS). After induction of anesthesia, an incision was made in the posterior-lateral chest wall and the cells were injected into the periosteum of the lower rib using a 27 gage needle; the wound was then closed with sutures. All mice were monitored for full recovery after anesthesia. Mice were examined daily and tumor size was measured three times/week using calipers. All animals underwent complete necropsy. For subsequent quantification of tumor volume, bioluminescent imaging was used.

### Ewing’s sarcoma cell culture and transfection

TC71 human ES cells were cultured as previously described (10, 11) to create luciferase-labeled tumor cells, the full-length firefly luciferase gene was spliced into the MigR1 expression vector and viral particles generated as previously described (12). TC71 cells were transduced on two consecutive days with infectious supernatant and then incubated for 48 h to allow for expression of incorporated retrovirus. Expression of EGFP, present as the second cistron in the expression cassette and translated under the direction of an internal ribosomal entry sequence, was confirmed via direct fluorescent microscopy. Transduced cells were purified via flow cytometry to select EGFP-expressing cells and luciferase expression was confirmed by visible light emission upon addition of luciferin.

### Isolation, characterization, and labeling of rat mesenchymal stem cells

Mesenchymal stromal cells were isolated from the bone marrow of Sprague-Dawley rats and expanded in MAPC media as previously described (13). Flow cytometric immunophenotyping was used to ensure the MSCs were CD11b−, CD45−, CD29+, CD49e+, CD73+, CD90+, CD105+, and Stro 14 1+. MSCs were labeled with the Qtracker 655 Cell Labeling Kit (Invitrogen, Carlsbad, CA, USA) per the manufacturer’s suggested protocol. Cell-labeling efficiency was >90% as confirmed by flow cytometry. Passage 3 cells were used for all experiments. For imaging in the mouse, the Qtracker signal was labeled red.

The rationale for using 1 × 10^6^ MSCs is based on previous experience with the use of MSCs in a non-tumor model. These amount of MSC cells were adequate to detect in all organs. Because it is technically easier to isolate rat MSCs, compared to mice, rat MSCs are used in this study.

### Delivery of MSCs

Mesenchymal stromal cells were removed from culture plates with 1X trypsin and washed with PBS. Next, the cells were suspended in PBS at a concentration of 1 million MSCs per milliliter of PBS and placed on ice until injection. After induction of anesthesia, the tail vein was cleansed with betadine and 1 million MSCs were injected. MSC injections were completed 1, 2, and 3 weeks after ES implantation. There were 15 mice in each group; one central group injected with PBS; one central group injected with lung and epithelial cells (CRL2300); and one treatment group.

### Bioluminescent imaging

After the injection of cells, the mice were imaged at different time points using an *in vivo*. IVIS 100 bioluminescence/optical imaging system (Xenogen, Alameda, CA, USA). d-Luciferin (Xenogen, Alameda, CA, USA) dissolved in PBS was injected intra-peritoneally at a dose of 150 mg/kg 10 min before measuring light emission. General anesthesia was induced with 5% isoflurane and five continued during the procedure. After acquiring photographic images of each mouse, luminescent images were acquired with various (1–60 s) exposure times. The resulting grayscale photographic and pseudocolor luminescent images were automatically superimposed by the IVIS Living Image (Xenogen) software to facilitate matching the observed luciferase signal with its location on the mouse. Regions of interest (ROI) were manually drawn around the bodies of the mice to assess signal intensity emitted. Luminescent signal was expressed as photons per second emitted within the given ROI. Tumor bioluminescence in mice linearly correlated with the tumor volume (14). Changes in tumor volumes were calculated by the ROI. Quantification used to calculate *p* values include Fisher exact test.

## Results

### Homing and engraftment of MSCs

After orthotopic Ewing’s chest wall tumors were established, MSCs were isolated, labeled, and delivered intravenously. Unexpectedly, the “naked” MSCs caused reduction in size of primary tumors and more so, lung metastasis.

**Figure 1:**
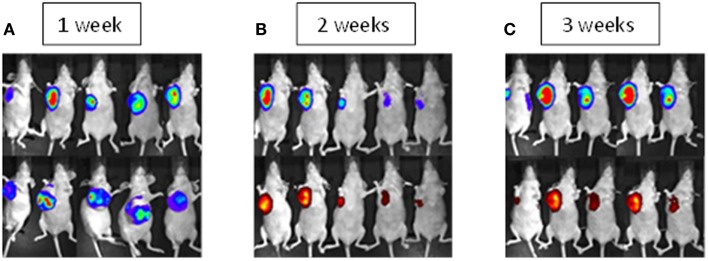
**MSC therapy in primary Ewing’s sarcoma**. The red color is labeled MSCs and the blue hues are labeled Ewing’s sarcoma TC71 cells. **(A)** Mice after the orthotopic implantation of human Ewing’s sarcoma cells into the rib to form chest wall tumors. **(B)** Mice 2 weeks after completion of two doses of intravenous mesenchymal stem cells (MSCs). The top panel shows a decrease in size of the primary chest wall tumors. The bottom panel indicates that the MSCs preferentially migrated to and engrafted at the site of chest wall tumors. **(C)** Mice 3 weeks after completion of three doses of intravenous MSCs where tumor starts to re-grow. The top panel shows no difference in primary tumor size when compared to control animals. The bottom panel indicates that the MSCs preferentially migrated to and engrafted at the site of chest wall tumors. Luciferase reporter from Ewing’s sarcoma cells show as intensity gradient from low to high cell load indicated by blue to red. Quantity of quantum dot labeled MSCs indicated by intensity of red color in bottom panel.

First, we show MSCs exclusively home to the site of the tumor. Figure 1 displays the primary chest wall tumor at 1, 2, and 3 weeks after three weekly consecutive MSC treatments. MSCs (red hues label the MSCs) home to the primary tumor site (blue hues indicate ES tumor) almost exclusively. At 1 week, 10 mice are seen with large chest wall tumors. At 2 weeks, the red hues show continued engraftment of the MSCs after two weekly injections in five mice. Also at 2 weeks, decreased tumor size can be seen in the chest wall tumor. At 3 weeks, chest wall tumor growth is stable.

**Figure 2:**
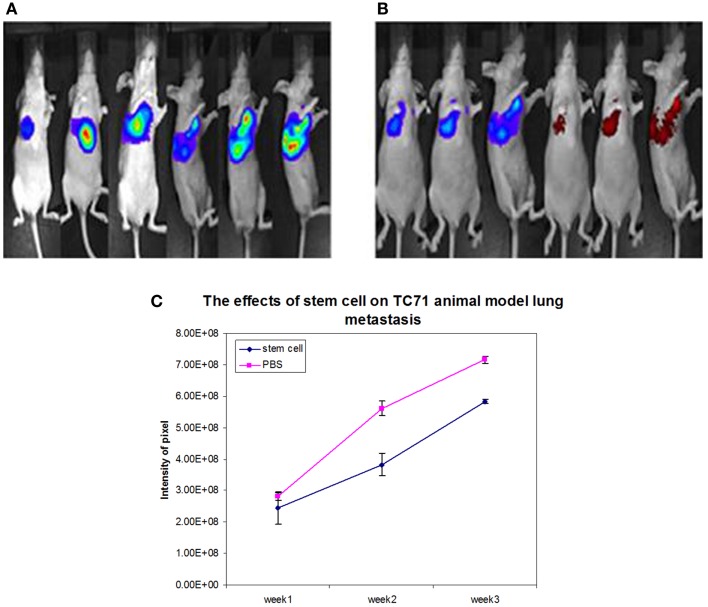
**MSC inhibition of metastatic Ewing’s sarcoma**. **(A)** Mice after the periosteal rib implantation of luciferase-labeled Ewing’s sarcoma cells (TC-T1) showing spontaneous bilateral pulmonary metastasis. **(B)** A decrease in luciferase-labeled Ewing’s sarcoma cells is observed up to 3 weeks after completion of intravenous MSC therapy as indicated by decreased blue signal. In addition, the injected MSCs were found to preferentially migrate to and engraft at the tumor sites as indicated by red intensity. **(C)** Quantification of the signal from the luciferase reporter shows a significant decrease in tumor burden 2 and 3 weeks after the completion of MSC therapy.

Figure 2 displays the lung metastasis pattern of tumor and MSC engraftment. These mice developed spontaneous bilateral lung metastasis. In Figure 2B, 3 weeks after MSC therapy, lung metastasis in the three mice pictured, has significantly decreased in size. Again as in the primary tumor, overlap of the red hues (MSCs) and blue hues (lung metastasis) demonstrate successful MSC engraftment. Slowing of lung metastatic growth in all six mice is quantified in Figure 2C.

**Figure 3:**
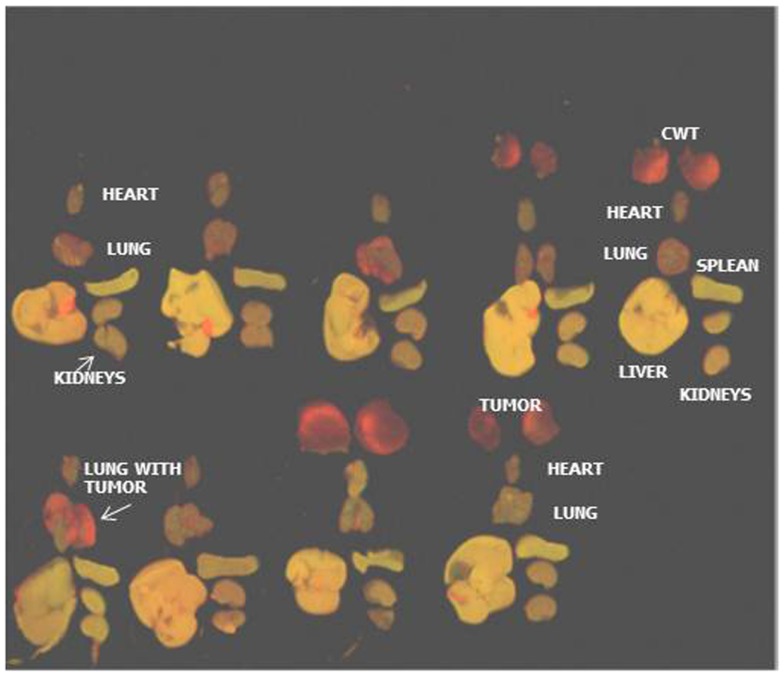
**MSCs home preferentially to the lung**. Tracking of the injected MSCs was completed by labeling the cells with quantum dots followed fluorescent imaging 6 h after injection. MSC injection was done after established chest wall tumors were 1 cm in size. The majority of MSCs remain within the lung parenchyma with very few found within the primary chest wall tumors. It is important to note that the MSCs remain within the lung in groups with or without pulmonary metastasis. This tracking experiment was repeated with lung epithelial cells (CRL2300) with similar results (cells remained sequestered in lung parenchyma with few found in primary chest wall tumors) (CWT, chest wall tumor; LM, lung metastasis).

We then wanted to identify the exact location microscopically of the MSCs in relationship to the primary tumor cells and MSCS in each organ. Organ explants from mice given MSCs were compared to mice in which lung epithelial cells (CRL2300) were delivered as controls. Figure 3 shows MSCs (green) distributed in lung with and without metastasis (f–i) with no MSCs seen in normal heart, kidney, liver, or spleen, at day 1 after treatment. Control animals were treated with labeled lung epithelial cells (CRL2300) (b–e). In contrast to the MSCs, few lung epithelial cells are seen in the lung without (b, d, e) or with (c) lung metastasis after intravenous (iv) delivery.

In animals with lung metastasis, after MSC delivery (f), MSCs infiltrate the lung parenchyma surrounding the tumor. No lung epithelial cells (d, e) and some MSCs (h, i) are seen infiltrating the primary tumors (red). Microscopically, the MSCs home to the lung metastasis more than the primary tumor.

### MSCs remain engrafted in the lung and decrease the incidence of pulmonary metastasis

**Figure 4:**
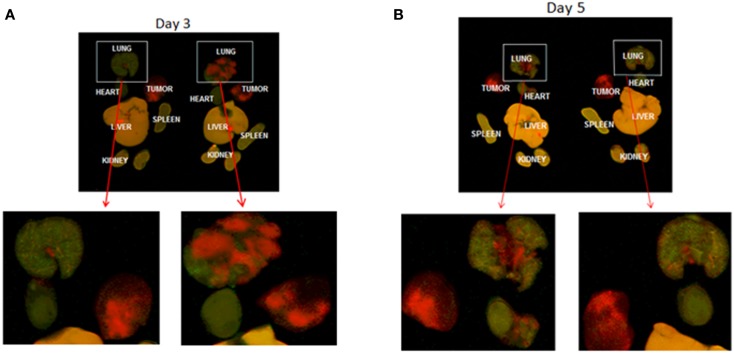
**Infrared whole mount images of mouse organs**. Red color indicates labeled tumor cells. MSCs are labeled green. Tumor = chest wall tumors from the rib injection site, which are completely red with no engrafted MSCs. Organs from two different mice on day 3 and day 5 after injection, are labeled. A-day 3 after MSC delivery. Red lung metastasis is seen in the top right image. Green MSCs are seen infiltrating the lung. The lung on the left does not have metastasis. **(B)** Day 5 – MSCs are seen engrafted in the lung with small tumors on the top left and no tumor on the top right. Red chest wall primary tumors do not show engraftment. We conclude from this that MSCs home to and engraft in lung but not primary tumor. Magnification of organs without metastasis and with metastasis from primary image **(A)**.

We then evaluated the longevity of MSC engraftment. Figure 4 shows animals at 3 and 5 days after iv delivery of MSCs. In Figure 4, labeled MSCs are seen in lung with and without lung metastases up to 5 days after injection. Few to no MSCs are seen in the primary tumor (c, d). Lung epithelial cells (CRL2300) were also injected as a non-specific cell line control and were also found to remain within the lungs 5 days after injection, but no tumor reduction was seen after injection of lung epithelial cells. Engraftment of MSCs are seen evenly distributed in lung without (c) and with (d) lung metastasis at day 3 and day 5.

### MSCs inhibit pulmonary metastasis in ES

**Figure 5:**
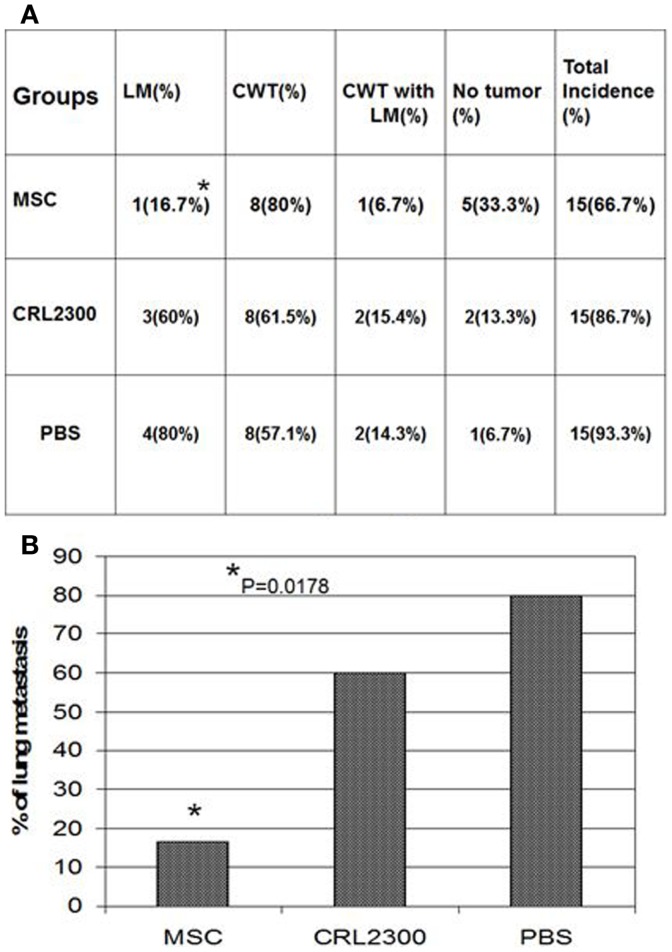
**MSC treatment significantly reduces lung metastasis in orthotopic xenograft model of Ewing’s sarcoma**. MSC treated mice compared to two control groups. Lung metastases are significantly fewer. MSC, mice treated with mesenchymal stem cells weekly for 3 weeks; CRL2300, lung epithelial cells, labeled the same as MSCs to serve as control; PBS, phosphate buffered saline injected group to serve as a second control group; LM, lung metastasis; CWT, chest wall tumor. Lung metastasis significantly decreased in size *p = 0.0178. **(B)** is a bar graph of data displayed in **(A)**.

Mesenchymal stromal cell therapy resulted in a decrease in the volume of lung metastasis after 3 weeks of treatment. Significantly, less tumor is seen in the lung metastasis of the mice treated with MSC therapy compared to those treated with control lung epithelial cells (Figure 5). Lung epithelial cells did not cause any observed decrease in pulmonary metastasis as seen with MSC therapy.

## Discussion

Preliminary research into the potential role of MSC therapy for ES has shown promise; (8) however, at the time of this manuscript, limited data has been published. Our ES model is unique in that it allows for the study of both primary chest wall tumors as well as pulmonary metastatic disease. MSCs have not previously been used to treat ES lung metastasis. Our model exploits, what are otherwise barriers, unique to the intravenous delivery of MSCs and afford an opportunity to give insight into the potential role of MSC therapy for ES metastasis. MSCs are known to sequester in the lungs after intravenous delivery. Our data show that the intravenous injection of MSCs in an ES model decreases the volume of pulmonary metastatic lesions. We found MSCs are found to preferentially remain in the pulmonary parenchyma and remain engrafted up to 5 days after delivery.

Fischer et al. have shown that the intravenous injection of MSCs results in a significant first-pass pulmonary effect leading to the majority of MSCs being sequestered within the lung parenchyma (7). Additionally, Harting et al. have shown that as few as 1.5% of MSCs bypass the pulmonary microvasculature after intravenous injection (15). While such a high rate of pulmonary sequestration poses a barrier to systemic treatment, it poses a unique opportunity for the treatment of the pulmonary metastatic disease associated with ES. Our data show that the majority of MSCs remain within the lung parenchyma and a decrease in pulmonary metastatic disease volume in MSC treated animals that is not observed with a non-tumorigenic cell line (CRL2300). Primary chest wall tumor size is not significantly affected by MSC therapy and shows rebound growth. The rebound effect we saw in the rib/chest wall tumors was very different than what we observed in the lung metastasis. The reason for this is unknown. We speculate, the differences in them microenvironment of the lung compared to the chest wall, and the activity of immune modulators and pro-inflammatory mediators, may affect these differences (16).

Since we are the first to show this effect of MSCs on lung malignancies, we plan more research on the etiology. Other authors have shown in carcinomas, MSCs can actually exacerbate tumor growth; specifically in breast carcinomas (17, 18). However, in gliomas, MSCs have been found to be therapeutic in reduction of tumor growth (19). The major difference is sarcomas are from mesenchymal origin and this may explain the potential therapeutic effect of MSCs on lung ES. Only one other group has found MSCs to have antitumorigenic effect sarcoma, in Kaposi’s sarcoma (20).

Mesenchymal stromal cells could be seen in the lung at 5 days after the injection. Since the MSCs were delivered weekly, we don’t know how long they would be present in the lung. In the future, we anticipate isolating the patient’s own MSCs from their bone marrow, and determining by Magnetic Resonance Imaging (MRI), how long they remain in the lung. Sequential MRI exams could also detect the effect of the MSCs in reduction or stabilization of lung metastasis.

It was unexpected that the “naked” MSCs were able to significantly decrease lung metastasis. This is the first time MSC treatment has been demonstrated to reduce lung metastasis or any other lung tumors. As a next step, we plan to transfect the MSCs with ES specific targeted therapy to afford further reduction in disease.

## Conclusions

The intravenous injection of MSCs offers a novel potential therapy for ES pulmonary metastasis. Our data show that the intravenous injection of MSCs in an ES model decreases the volume of pulmonary metastatic lesions. This is the first description of successful MSC therapy of lung tumors.

## Conflict of Interest Statement

The authors declare that the research was conducted in the absence of any commercial or financial relationships that could be construed as a potential conflict of interest.
